# Olive Mill Wastewater: A Sustainable Natural Solution for Nitrate Contamination Control in Agricultural Systems and Quality Enhancement of Multileaf Lettuce Cultivation

**DOI:** 10.1021/acssuschemeng.6c02426

**Published:** 2026-04-27

**Authors:** Adrián Hernández Fernández, Eduardo Iniesta Lopez, Yolanda Garrido, Ana Sánchez Zurano, Antonia Pérez de los Ríos, Francisco José Hernández Fernández

**Affiliations:** † Department of Chemical Engineering, 16751 University of Murcia, Murcia 30100, Spain

**Keywords:** Agro-industrial byproducts, biological nitrification inhibition, nitrogen leaching, circular economy, *Lactuca sativa* L.

## Abstract

The search for sustainable alternatives to synthetic nitrification inhibitors (SNIs) is critical for reducing agricultural nitrogen losses and environmental impact. This study assessed the effects of low concentrations (5, 10, and 50 mg phenolics•L^–1^) of olive mill wastewater (OMW) soluble fraction on nitrogen dynamics, phytotoxicity, and quality traits of *Lactuca sativa* L. cultivated under controlled soil conditions simulating field conditions. Treatments included a positive control with ammonium fertilization (Control P) and Dicyandiamide (DCD) as an SNI treatment (P+DCD 25 mg/L). P+DCD 25 mg/L and P+OMW 50 mg/L reduced nitrate (NO_3_^–^) leaching by 81% and 65% and nitrite (NO_2_^–^) leaching by 93% and 54%, respectively, compared to Control P (*p* < 0.05). However, the former increased ammonium (NH_4_^+^) leaching by 59%, while the latter reduced it by 34%. As a result, total inorganic nitrogen leaching was similarly reduced under both treatments—61% and 58%, respectively. Lower concentrations of the OMW soluble fraction had no significant effect on nitrogen dynamics or crop performance. At 50 mg phenolics/L, the OMW soluble fraction enhanced lettuce growth and fresh biomass by over 50% compared to Control P, without phytotoxic effects, and preserved leaf morphology and coloration within the typical ranges reported for *Lactuca sativa* L. under optimal cultivation. Leaf NO_3_^–^ contents remained below EU regulatory limits in all treatments. Also, significant matrix interferences were detected during HPLC-UV/vis quantification of NO_2_^–^ in lettuce tissues, highlighting the need for complementary analytical methods in leafy vegetables. These findings support the valorization of agro-industrial byproducts within a circular economy approach to improve nitrogen use efficiency and crop quality in sustainable horticultural systems.

## Graphical abstract

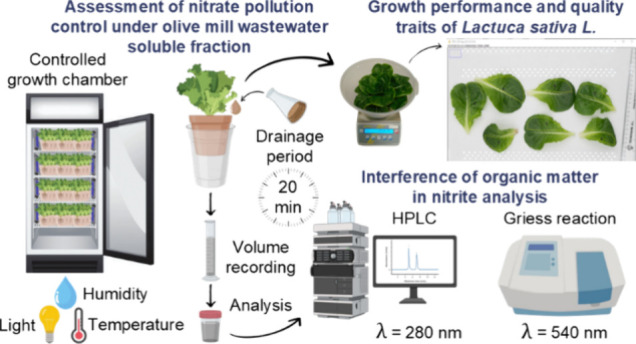

## 1 Introduction

The increasing demand for healthy, convenient food options has driven substantial growth in the ready-to-eat fresh produce sector, particularly for leafy vegetables.^1^ Minimally processed leafy greens have emerged as a key product category, offering convenience while preserving essential nutritional qualities, through limited processing steps—typically involving harvesting, washing, drying, and packaging under modified atmospheres, followed by cold storage.^2,3^ Among these, “multi leaves”—mature leaves harvested at reduced sizes due to dense planting—are especially valued for their suitability for direct consumption without the need for further cutting.^4^

These vegetables contribute essential nutrients such as fiber, minerals, vitamins, and bioactive compounds to the human diet.^2,5^ Their quality is strongly influenced by preharvest conditions, with water management and the application of chemical agents during cultivation playing particularly significant roles.^6,7^ Recent studies in lettuce also support the strong influence of preharvest treatments on crop performance and quality-related traits. For example, exogenous melatonin has been reported to improve lettuce growth, antioxidant responses, and photosynthetic pigments under salinity stress,^8^ whereas moringa-derived products have shown potential as natural nutrient sources to enhance lettuce growth while reducing nitrate accumulation under organic production conditions.^9^ Other factors such as the salinity of irrigation water, the timing of harvest, and rapid postharvest cooling are also critical for preserving quality and prolonging shelf life.^10–12^

The production of leafy vegetables in the Region of Murcia in Spain plays a key role in the local economy. However, its proximity to the Mar Menor—a lagoon severely impacted by pollution and eutrophication—highlights the urgent need to implement sustainable agricultural practices.^13,14^ Balancing productivity with environmental protection is essential for the long-term viability of agriculture in this ecologically sensitive area. Developing innovative solutions that reduce the environmental footprint of crop production, while safeguarding ecosystem integrity, is thus imperative to preserve both economic and environmental sustainability.^15^

One of the most pressing environmental concerns is NO_3_^–^ pollution from agricultural sources. Nitrification, driven by soil bacteria, converts NH_4_^+^ into NO_3_^–^, a highly soluble form of nitrogen (N) prone to leaching.^16^ This not only reduces N availability in the soil but also promotes NO_3_^–^ leaching into groundwater, contributing to nutrient pollution and eutrophication. In vulnerable areas like the Mar Menor, this has led to major ecological degradation, including loss of aquatic biodiversity and deterioration of fisheries, placing further pressure on a regional economy dependent on agriculture and tourism.^13,14^

Given these environmental challenges, there is a crucial need to develop effective mechanisms for controlling nitrification. Traditional approaches have relied on SNIs, such as DCD, to mitigate N losses.^17^ However, while effective, these synthetic compounds carry risks, as they can be absorbed by plants, potentially exposing consumers to chemical residues in leafy vegetables, where such compounds rapidly migrate to edible parts.^18,19^ This highlights the need to explore natural, biodegradable alternatives to SNIs, especially in organic farming where chemical inhibitors are not permitted. Studies have shown that certain plant extracts can inhibit nitrification while promoting sustainable agricultural practices.^20,21^

In this context, valorization of agro-industrial byproducts aligns with circular economy principles, offering a strategy to reduce waste while improving agricultural sustainability. Olive oil production generates large volumes of byproducts rich in bioactive phenolics, which have demonstrated biological activity, including potential for nitrification inhibition.^22,23^ Their use can improve soil health and reduce dependence on synthetic inputs, contributing to resource efficiency and climate resilience, particularly in Mediterranean regions like Murcia.

Our research group has previously investigated the efficacy of olive mill wastewater (OMW) soluble fraction as a nitrification inhibitor under in vitro conditions and in soil devoid of plants. We have also documented its biodegradability and analyzed its composition in terms of macro- and micronutrients beneficial for crops.^24,25^ The use of OMW soluble fraction may provide a dual advantage: mitigating N losses while supplying essential nutrients to plants, representing an innovative step toward sustainable agricultural practices. Nonetheless, previous studies substantiate that byproducts from the olive oil industry can act as effective herbicides, highlighting the need to evaluate the implications of their use in the agriculture of leafy vegetables.^22,26^

Therefore, this study aims to evaluate the agronomic and environmental implications of applying OMW soluble fraction in soils cultivated with multileaf lettuce. Three phenolic concentrations of OMW soluble fraction (50, 10, and 5 mg phenolics/L) were tested and compared with DCD at 25 mg/L, a standard synthetic inhibitor in European agriculture. A control treatment without inhibitor was included. We assessed the concentrations of NO_3_^–^, NO_2_^–^, NH_4_^+^, total nitrogen (TN), and organic nitrogen (ON) in soil and leachates, as well as NO_3_^–^ and NO_2_^–^ levels in lettuce leaves. In addition, we evaluated lettuce biomass and key quality parameters such as morphology and color. Additionally, we examined potential analytical interferences in NO_2_^–^ quantification in plant extracts, using the HPLC method described by Kyriacou et al.^27^ Clarifying these interferences is key to ensuring accurate NO_2_^–^ determination in future research and applications.

## 2 Materials and Methods

### 2.1 Physicochemical Characterization of OMW Soluble Fraction

The OMW soluble fraction used in this study was obtained following the procedure previously described by Hernández et al.^23^ Briefly, 40 mL of OMW were centrifuged at 3000 × g for 25 min at 4 °C. The supernatant was then filtered through a 0.45 μm polypropylene membrane and stored at 4 °C until analysis and subsequent dilution to the target concentrations used as biological nitrification inhibitor (BNI) treatments.

Total phenolic content (TPC) was determined by the Folin-Ciocalteu method described by Cicco et al.,^28^ with minor modifications. In brief, 300 μL of appropriately diluted sample were mixed with 300 μL of Folin-Ciocalteu reagent and allowed to react for 2 min. Then, 2.4 mL of 5% sodium carbonate solution were added, and the mixture was incubated for 1 h in the dark at room temperature. Absorbance was measured at 760 nm using a T80 UV–vis spectrophotometer (PG Instruments, United Kingdom). Results were expressed as mg gallic acid equivalents per liter (mgGAE•L^–1^).

The main phenolic compounds in the same OMW soluble-fraction batch, namely hydroxytyrosol, tyrosol, and oleuropein, were quantified by reverse-phase HPLC-DAD at 280 nm using syringic acid as internal standard, under the chromatographic conditions described by Hernández et al.^23^

Phosphorus (P) and potassium (K) contents were determined by ICP-MS (Agilent 7900, Agilent Technologies, USA) following Fernández et al.^24^ Calibration curves were prepared from certified multielement standards (High-Purity Standards; Agilent Technologies) using concentration ranges of 0–100 mg•L^–1^ for P and 0–10000 mg•L^–1^ for K, in 2% (v/v) HNO_3_.

Total nitrogen (TN) and total organic carbon (TOC) were determined according to ISO 20236 by catalytic high-temperature combustion using a multi N/C 3100 analyzer (Analytik Jena, Germany). Based on these measurements, the TOC/TN mass ratio of the OMW soluble fraction was calculated. In addition, NH_4_^+^, NO_2_^–^, and NO_3_^–^ concentrations in the OMW soluble fraction were determined using the same analytical procedures described below for liquid samples.

All physicochemical parameters reported in Table 1 were determined in triplicate from the same OMW soluble-fraction batch.

**Table 1:** Physicochemical Properties of the OMW Soluble Fraction Used in This Study^a^

Parameter	Conc. ± SD	Unit
P	792 ± 6	mg•L^–1^
K	9532 ± 83	mg•L^–1^
NH_4_^+^	<DL	mg•L^–1^
NO_2_^–^	<DL	mg•L^–1^
NO_3_^–^	<DL	mg•L^–1^
TN	914.7 ± 5.8	mg•L^–1^
TOC	38814 ± 923	mg•L^–1^
TOC/TN	42.4 ± 1.1	-
TPC	5340 ± 84	mgGAE•L^–1^
Hydroxytyrosol	1661 ± 101	mg•L^–1^
Tyrosol	553 ± 27	mg•L^–1^
Oleuropein	<DL	mg•L^–1^

a TN: total nitrogen; TOC: total organic carbon; TPC: total phenolic compounds. Values are expressed as mean ± standard deviation of three analytical replicates (n = 3) obtained from the same batch of OMW soluble fraction. DL: detection limit.

### 2.2 Plant Material and Growing Conditions

Cos-type lettuce plants (*Lactuca sativa* L. var. *longifolia*) at commercial transplant size were provided by the nursery Semilleros del Sureste S.L. (southeastern Spain). The growth substrate was prepared by mixing sieved natural agricultural soil (2 mm) with river sand and vermiculite in a 50:25:25 volumetric ratio. The physicochemical characterization of the unfertilized soil substrate used in this study is presented in Table 2. One-liter plastic pots (12 cm top diameter, 8 cm base, 11 cm height) were filled with 690 g of the prepared soil mixture, with one plant grown per pot. Fertilizers—1.07 g (NH_4_)_2_SO_4_, 0.314 g K_2_SO_4_, and 0.407 g of 18% superphosphate—were thoroughly mixed into the substrate, corresponding to 200 kg N•ha^–1^, 70 kg P_2_O_5_•ha^–1^, and 150 kg K_2_O•ha^–1^.

**Table 2:** Physicochemical Characterization of the Unfertilized Soil Substrate Used in the Present Study^a^

Parameter	Value	Unit
Type of soil	Sandy loam	-
pH	7.71 ± 0.02	-
EC	0.37 ± 0.06	dS•m^–1^
TOC	51.28 ± 0.98	g•kgDW^–1^
TN	399.40 ± 11.37	mg N•kgDW^–1^
NH_4_^+^	0.36 ± 0.49	mg NH_4_^+^•kgDW^–1^
NO_3_^–^	13.21 ± 5.72	mg NO_3_^–^•kgDW^–1^
NO_2_^–^	1.41 ± 0.23	mg NO_2_^–^•kgDW^–1^

a Values are presented as mean ± standard deviation of three analytical replicates (n = 3).

To assess the effect of different nitrification inhibitors on planted systems, four treatments were applied in distilled water: OMW soluble fraction at 50, 10, and 5 mg phenolics/L (P+OMW 50 mg/L, P+OMW 10 mg/L, P+OMW 5 mg/L), and DCD at 25 mg/L (P+DCD 25 mg/L) as a synthetic reference. These treatments were compared with planted control pots (Control P) and unplanted soil pots (Control S). Treatments were arranged in a randomized complete block design with three biological replicates (n = 3), each block containing one pot of every treatment. Pot positions within the growth chamber were randomized to minimize possible spatial effects. Prior to treatment initiation, all pots received irrigation with distilled water.

Experiments were conducted in a controlled growth chamber (FITOCLIMA S600 PLH, ARALAB) set at 23/18 °C (day/night), with a 16/8 h light/dark photoperiod and 200 μmol•m^–2^•s^–1^ of photosynthetically active radiation. Lettuce seedlings were individually transplanted into pots and initially irrigated to field capacity using distilled water. Soil moisture was subsequently maintained above 70% field capacity throughout the experiment. All plants received distilled water at 3 days after transplanting (DAP), and the application of the nitrification inhibitor treatments began at 5 DAP. Weekly irrigation volumes were adjusted to plant growth, set at 21, 28, 32, and 35 L•m^–2^ for weeks one through four, respectively, over a total cultivation cycle of 31 days. Treatments were applied manually via localized irrigation four times per week, targeting the base of each plant and avoiding foliar contact.

### 2.3 Determination of NH_4_^+^, NO_2_^–^, NO_3_^–^, and TN in Leachates, Soil, and Lettuce Tissues

Nitrogen forms were analyzed as NH_4_^+^, NO_3_^–^, and NO_2_^–^ in both leachates and soil extracts, while TN was determined in leachates and solid soil samples. In lettuce tissues, only NO_3_^–^ and NO_2_^–^ were quantified in leaf extracts. ON in leachates was estimated as the difference between TN and the sum of inorganic forms (NH_4_^+^, NO_2_^–^, and NO_3_^–^).

NH_4_^+^ concentration was analyzed using the APHA 4500-NH_3_ colorimetric method^29^ with a Spectroquant Prove 300 spectrophotometer (Merck, Germany).

NO_2_^–^ and NO_3_^–^ concentrations were determined by HPLC, following the method described by Kyriacou et al.,^27^ using an Agilent 1220 Infinity Series chromatograph equipped with a diode array detector (G4294B, Agilent Technologies, USA). Separation was achieved with a Zorbax Eclipse XDB-C18 column (4.6 × 250 mm, 5 μm) at 30 °C, using a 0.01 M octyl-ammonium phosphate buffer (prepared in 20% methanol and adjusted to pH 6.5 with phosphoric acid) as the mobile phase. The method employed a 10 μL injection volume at a 1.0 mL•min^–1^ isocratic flow, with detection at 220 and 275 nm. Under these conditions, retention times for NO_2_^–^ and NO_3_^–^ were 6.9 and 8.5 min, respectively. Quantification was performed using external calibration curves.

Additionally, NO_2_^–^ was quantified using the Griess colorimetric method, following the APHA 4500-NO_2_.^29^ Absorbance measurements were performed at 540 nm with a T80 UV–visible Spectrophotometer (PG Instruments Limited, United Kingdom). The use of both HPLC and Griess methods for NO_2_^–^ quantification enabled the assessment of potential interferences caused by organic matter.

TN in leachates was measured following the ISO 20236 method, based on catalytic oxidative combustion at high temperature, using an Analytik Jena multi N/C 3100 analyzer (Analytik Jena, Germany). TN in solid soil samples was determined according to ISO 13878 (dry combustion) using a LECO CN828 (LECO Corporation, USA).

### 2.4 Sampling of Leachates, Soil Extracts, Soil, and Lettuce Tissues

For leachate collection, pots were placed on individual plastic cups. After each irrigation event, a 20 min drainage period was allowed, and the leachate volume was recorded and collected in plastic containers stored at 4 °C until analysis. Only the initial saturation irrigation and the first control irrigation were analyzed individually. The remaining leachates from each week (four irrigation events) were pooled to reduce the number of analyses while representing all drained leachate. All leachate samples were filtered through 0.45 μm polypropylene filter prior to analysis. For each sampling date, NH_4_^+^, NO_2_^–^, NO_3_^–^, TN and ON in leachates were expressed as mg•m^–2^ of soil surface using the equation:

$$Conta\min ant\;in\;leachate\;\left(mg\cdot m^{-2}\right)=\frac{\left[Concentration\times Volume\right]}{Area}$$ 1

where “Concentration” is the concentration of the target compound in the sample (mg•L^–1^), “Volume” is the total volume of leachate collected on that date (L), and “Area” is the surface area of the soil in the pot (m^2^).

The cumulative leaching of each N species throughout the trial was estimated by summing, for all replicates and sampling events, the product of its measured concentration and the corresponding leachate volume, divided by the soil surface area of each pot, according to the following expression:

$$Cumulative\;conta\min ant\;in\;leachate\;\left(mg\cdot m^{-2}\right)=\overset{n}{\underset{i=5}{\sum }}\left(\frac{C_{i}\times V_{i}}{A}\right)$$ 2

where “C_i_” is the concentration (mg·L^–1^) of the N species analyzed (NH_4_^+^, NO_2_^–^, NO_3_^–^, TN or ON), “V_i_” is the leachate volume collected (L), and “A” is the surface area of the pot (m^2^) at sampling event “i”. The summation was carried out from the fifth day after planting (DAP 5) to the end of the experimental period (DAP 31).

NO_3_^–^ and NO_2_^–^ in lettuce tissues were analyzed using a modified version of the protocol by Kyriacou et al.^27^ All lettuce leaves were frozen in liquid nitrogen and homogenized using a mixer grinder Blixer 2 (Robot Coupe, France). Then, 5 g of homogenate were placed in 50 mL centrifuge tubes with 30 mL of ultrapure water. The mixture was vortexed for 2 min, sonicated at 80 °C for 20 min, tempered for 20 min, vortexed again for 2 min, and filtered through filter paper into a 50 mL volumetric flask, which was filled to volume with ultrapure water. The solution was subsequently filtered through a 0.45 μm filter and stored at 4 °C until analysis. Leaf samples were analyzed at 5 and 31 DAP. Nitrogen contents were expressed as mg•kg^–1^ fresh weight (FW) of the lettuce.

Soil extracts for NH_4_^+^, NO_3_^–^, and NO_2_^–^ determination were prepared using a modified protocol based on the Sigma-Aldrich analytical methods.^30–32^ Briefly, 25 g of moist, stone-free soil were mixed with 50 mL of 0.025 mol•L^–1^ CaCl_2_ in a glass bottle and shaken for 1 h at 25 °C and 125 rpm in a ventilated orbital incubator SELECTA 3000957 (J.P. SELECTA, Spain). After shaking, 20 mL of the suspension were centrifuged at 2500 × g for 25 min at 20 °C. The supernatant was filtered through a 0.45 μm polypropylene filter and stored at 4 °C until analysis. An additional soil sample was oven-dried at 70 °C for 96 h to determine moisture content.

For TN analysis in solid soil samples, 30 g of soil were air-dried at room temperature, ground with a mortar, and sieved through a 200 μm mesh. Soil samples were collected at the start of the treatment with nitrification inhibitors (5 DAP) and after lettuce harvest (31 DAP). Nitrogen contents were expressed as mg•kg^–1^ dry weight (DW).

### 2.5 Lettuce Growth and Color

The effect of nitrification inhibition treatments on lettuce growth was evaluated by recording the fresh weight of shoot and the number of leaves per plant (in all plants), immediately after harvest. Then, all the leaves of the lettuce were placed individually with the adaxial surface facing up on a tray and transferred into a photographic box for image capture for morphological characteristics analysis. Morphological characteristics as well as color attributes of the leaves were assessed through digital image analysis as described by García et al.^33^ with modifications. Leaf images were captured using a Nikon D7100 digital camera mounted above a photography studio box (HPB-80XD) measuring 80 cm × 80 cm × 80 cm (height × length × width), with matte white translucent walls and a black matte ground. Illumination was provided by four LED strips positioned horizontally on the inner top of the box. The color temperature of the light was 5500K, and the luminous flux was 26,000 lm. Image acquisition was conducted in a darkroom at room temperature, and all images were captured with a graduated ruler at the same level as the sample for reference. On each evaluation date, an image of a gray card was captured under identical conditions for later gray balance correction of the other images. All images were saved in RAW format (5472 × 3648 pixels) with the sRGB color space.

The images were converted to the HSB color space (hue, saturation, brightness on a 0–255 scale), and the leaf shape parameters were evaluated using the open-source image processing software ImageJ 1.50i (NIH, Bethesda, MD, USA). The following morphological parameters were analyzed: whole leaf area, leaf length and width, and roundness, which was calculated using the formula:

3 $$Roundness=\frac{Perimeter^{2}}{4\pi \times Area\times 1.064}$$

Values of roundness closer to unity indicate a more circular shape.

After imaging, the leaves were placed in a freezer bag and stored at −20 °C until further chemical analysis.

### 2.6 Statistical Analysis

All data were analyzed with PASW Statistics 28 for Windows (SPSS Inc., USA). A one-way analysis of variance (ANOVA) was utilized to determine significant differences among the groups, with a significance threshold established at *p* < 0.05. When significant differences were identified, post hoc comparisons were carried out using Tukey’s honestly significant difference (HSD) test. Furthermore, bilateral correlations between the variables were assessed through Pearson’s correlation coefficient, maintaining a 95% confidence interval.

## 3 Results and Discussion

### 3.1 Assessment of Organic Matter Interference in NO_3_^–^ and NO_2_^–^ Determination Across Leachate, Soil Extract, and Lettuce Tissue Matrices

NO_3_^–^ and NO_2_^–^ concentrations were quantified by HPLC-UV/vis detection, following the method of Kyriacou et al.,^27^ in leachates, soil extracts, and lettuce leaf extracts. To assess possible interference from organic compounds, absorbance was monitored at 220 nm—commonly used for NO_3_^–^/NO_2_^–^ detection—and at 275 nm, a wavelength associated with the presence of chromophoric groups derived from organic matter, which can cause interference in spectrophotometric assays.^34–36^ This dual-wavelength approach was based on previous findings where overestimation of NO_3_^–^ and NO_2_^–^ occurred in leachates from soils treated with highly concentrated OMW as BNI (>250 mg phenolics•L^–1^), but not at lower concentrations (50 mg phenolics•L^–1^).^25^

Previous studies have employed HPLC-UV/vis for NO_3_^–^ and NO_2_^–^ quantification in plant matrices.^37,38^ Croitoru et al.^37^ quantified NO_2_^–^ in beetroot at 520 nm after derivatization, while Özdestan et al.^38^ used direct detection at 205 nm to measure both analytes in ten leafy vegetables, including *Lactuca sativa L.*, where NO_2_^–^ was not detected. However, no previous work has reported interference from organic matter in HPLC-UV/vis-based NO_2_^–^ quantification at 220 nm in *Lactuca sativa* or similar leafy vegetables.

Chromatograms shown in Figure 1 correspond to the Control P treatment. In this treatment, no interference at 275 nm was detected in any matrix for NO_3_^–^, confirming the reliability of HPLC-UV/vis for NO_3_^–^ quantification under the tested conditions (Figure 1E, 1G, 1I). Similarly, no interference was observed for NO_2_^–^ in leachates and soil extracts (Figure 1F, 1H). These results are consistent with previous findings in soils amended with OMW soluble fraction as a BNI, where no measurable interference was reported for either NO_3_^–^ or NO_2_^–^ when using OMW soluble fraction containing low concentrations of phenolic compounds.^25^ However, in lettuce leaf extracts, a coeluting peak at the NO_2_^–^ retention time showed absorbance at 275 nm equivalent to 95% of that at 220 nm (Figure 1D), indicating strong interference from plant-derived organic matter, despite the low NO_2_^–^ concentrations measured in the analyzed leaf extracts (∼1 mg•L^–1^). This pattern was consistent across treatments.

**Figure 1:**
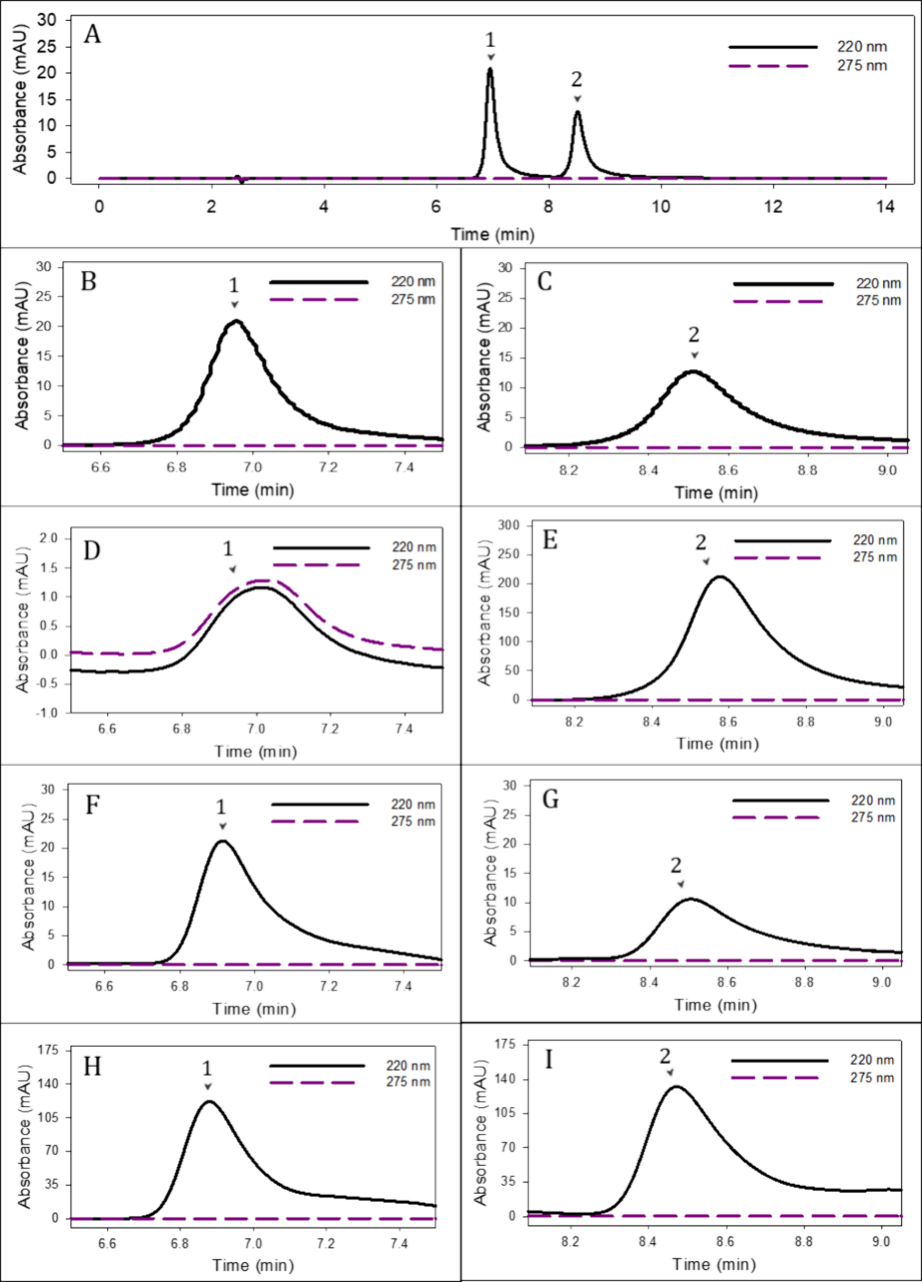
HPLC chromatograms of the reference standards for NO_2_^–^ and NO_3_^–^ (A, B, C), compared with samples from the control plant treatment (Control P), including lettuce extract (D, E), soil extracts (F, G), and leachate water (H, I). Signals were recorded simultaneously at 220 nm (solid line) and 275 nm (dashed line). Identified peaks correspond to (1) NO_2_^–^ and (2) NO_3_^–^.

Given the observed interference, NO_2_^–^ concentrations were also determined using the colorimetric Griess assay, following APHA Standard Method 4500-NO2, a protocol widely recognized by regulatory agencies for NO_2_^–^ quantification in water and food matrices.^39^ Correlation between methods was high for leachates (r = 0.995) and soil extracts (r = 0.959), with no significant differences (*p* > 0.05) in NO_2_^–^ concentrations. In contrast, results for lettuce tissue extracts showed low correlation (r = 0.531) and significant differences (*p* < 0.05) in NO_2_^–^ quantification, likely due to matrix interference, potentially related to species or cultivar-specific plant metabolites and low analyte levels near the detection limit.

These findings highlight a previously unreported limitation of HPLC-UV/vis for NO_2_^–^ detection in leafy vegetables, particularly *Lactuca sativa L.*, underscoring the need for additional validation steps, including sample cleanup strategies such as SEP-PAK and systematic comparison with the Griess method. Accordingly, NO_2_^–^ concentrations reported in this study for all matrices were those obtained with the Griess method.

### 3.2 Effect of Synthetic and Natural Nitrification Inhibitors on N Dynamics in Agricultural Soils Cultivated with Leafy Vegetables

Unlike previous studies conducted under in vitro conditions^24^ or in bare soil systems,^25^ this study evaluates the impact of soluble OMW fractions on N dynamics in agricultural soils cultivated with *Lactuca sativa L.*, a representative leafy vegetable known for its sensitivity to chemical compounds. Three OMW soluble fraction treatments were applied at low phenolic concentrations (P+OMW 50, 10, and 5 mg•L^–1^), alongside the synthetic nitrification inhibitor Dicyandiamide (P+DCD 25 mg•L^–1^), a widely used reference compound within legal limits (Spanish Decree-Law 999/2017). These phenolic concentrations were selected based on previous findings, where higher doses (≥250 mg•L^–1^) not only caused nitrification inhibition but also promoted the immobilization of inorganic nitrogen by heterotrophic soil microorganisms, driven by the high labile carbon content and elevated C/N ratio (∼40) of the OMW soluble fraction, ultimately reducing inorganic N potentially available for plant uptake.^25^ Additionally, two control conditions were included: Control S and Control P, which served as baseline references to estimate the nitrification inhibition potential of each treatment—through relative reductions in NO_3_^–^ and NO_2_^–^ concentrations in leachates—as well as to evaluate the potential for increased NH_4_^+^ retention in soil. By including a crop in the experimental setup, this strategy enables a more accurate assessment of the treatments’ effects on N availability and their contribution to improving nitrogen use efficiency (NUE) under realistic cultivation conditions, effectively bridging the gap between controlled soil assays and field-scale applications. In particular, the use of *Lactuca sativa L*. allows simultaneous monitoring of N dynamics and potential phytotoxicity, as this species has been reported as a reliable bioindicator for chemical stress.^40–42^ Additionally, while OMW has demonstrated herbicidal activity at high phenolic concentrations,^26^ its agronomic value at lower doses—due to its content of macro- and micronutrients—remains an area of interest for sustainable fertilization practices.^22^

This work aims to assess the dual functionality of diluted OMW soluble fractions: their ability to regulate the N cycle while avoiding adverse effects on crop development. Through a comparative analysis with DCD, the study contributes to the identification of viable natural alternatives to synthetic inhibitors. This approach supports more sustainable fertilization strategies and promotes circular economy principles by valorizing OMW, a major agro-industrial byproduct in Mediterranean regions.

#### 3.2.1 Nitrogen Retention and Transformation in Soil

In Control S, a typical nitrification pattern was observed by 31 DAP, characterized by substantial NH_4_^+^ depletion and a significant accumulation of NO_3_^–^ in the soil (Table 3), consistent with rapid microbial oxidation under aerobic conditions.^43^ Initial NO_2_^–^ concentrations were moderate but declined to nearly undetectable levels, likely due to oxidation to NO_3_^–^ or leaching. Minimal changes in ON were recorded, suggesting limited mineralization. TN losses were primarily linked to NO_3_^–^ leaching, with minor contributions from NH_4_^+^ and NO_2_^–^. The limited mobility of NH_4_^+^ is expected due to its retention via cation exchange with negatively charged sites in the soil’s humic-clay complex.^44^

**Table 3:** Levels (mg•kg^–1^ dry weight (DW)) of Different Nitrogen Species (NO_3_^–^, NO_2_^–^, NH_4_^+^, ON, and TN) in Soil Samples^a^

Days after planting	Treatment	Nitrate (mgNO_3_^–^•kgDW^–1^)	Nitrite (mgNO_2_^–^•kgDW^–1^)	Ammonium (mgNH_4_^+^•kgDW^–1^)	Organic nitrogen (mgN•kgDW^–1^)	Total nitrogen (mgN•kgDW^–1^)
5	Control S	18.61 ± 5.76	15.91 ± 4.11 B	144.70 ± 28.70	378.6 ± 49.0	500.00 ± 43.59
	Control P	20.08 ± 3.10 NS	25.20 ± 0.96 A	127.77 ± 15.49 NS	371.9 ± 19.5 NS	483.33 ± 15.28 NS
31	Control S	82.18 ± 1.79 a	0.47 ± 0.03 a	2.85 ± 1.35 b	369.1 ± 10.1 a	390.00 ± 10.00 a
	Control P	10.77 ± 6.54 b	0.31 ± 0.01 a	1.28 ± 1.12 b	319.8 ± 41.7 b	323.33 ± 41.63 b
	P+DCD 25 mg L^–1^	3.58 ± 0.87 b	0.01 ± 0.01 b	12.21 ± 5.11 a	353.0 ± 25.5 ab	363.33 ± 25.17 ab
	P+OMW 50 mg L^–1^	6.36 ± 0.26 b	0.21 ± 0.01 a	0.17 ± 0.15 b	331.7 ± 22.7 b	333.33 ± 22.73 b
	P+OMW 10 mg L^–1^	8.46 ± 3.16 b	0.37 ± 0.13 a	0.18 ± 0.16 b	324.5 ± 25.1 b	326.67 ± 25.13 b
	P+OMW 5 mg L^–1^	5.00 ± 0.38 b	0.38 ± 0.05 a	0.16 ± 0.14 b	333.6 ± 15.0 b	335.00 ± 15.00 b

a Recorded at the beginning and the end of the trial, in pots with cultivated lettuce irrigated with different nitrification inhibitor treatments: DCD at 25 mg/L (P+DCD) and OMW soluble fraction at 50, 10, and 5 mg of phenolic content/L (P+OMW), compared with control plants (Control P) and pots with soil but without plants (Control S) irrigated with distilled water. Values represent the mean ± standard deviation of 3 replicates. Values with different letters in the same column are significantly different (*p* < 0.05) according to Tukey’s HSD test, with capital letters and lowercase letter for day 5 and day 31 analysis, respectively. Ns, not significant.

In Control P, a similar transformation pattern was observed, but plant presence markedly influenced N dynamics. NH_4_^+^ was fully depleted by 31 DAP, and NO_2_^–^ concentrations dropped below detection limits, indicating efficient nitrification and/or plant uptake (Table 3). Final soil NO_3_^–^ levels were significantly lower than in Control S (*p* < 0.05), likely reflecting plant absorption during growth. A significant decrease in ON (*p* < 0.05) was also detected, potentially driven by plant-induced depletion of inorganic N pools, which may have stimulated mineralization. This ON decline was not reflected in increased leached ON (Table 4), suggesting its transformation into NH_4_^+^ and subsequent nitrification or leaching. Overall, TN retention in soil was significantly lower than in Control S (*p* < 0.05), reflecting both plant uptake and enhanced mineralization, as expected under fertilized, irrigated cropping systems.

**Table 4:** Total Input (mg•m^–2^) of Different Nitrogen Species (NO_3_^–^, NO_2_^–^, NH_4_^+^, ON, and TN) in Leachate Water Throughout the Entire Trial in Pots with Cultivated Lettuce, Irrigated with Different Nitrification Inhibitor Treatments^a^

Treatment	Nitrate (mgNO_3_^–^•m^–2^)	Nitrite (mgNO_2_^–^•m^–2^)	Ammonium (mgNH_4_^+^•m^–2^)	Organic nitrogen (mgN•m^–2^)	Total nitrogen (mgN•m^–2^)
Control S	26819.8 ± 1047.0 a	5659.5 ± 756.1 a	2796.9 ± 569.4 a	–11.7 ± 712.1	9941.6 ± 450.2 a
Control P	17395.6 ± 4124.0 b	3972.2 ± 710.7 b	1254.1 ± 276.6 c	9.4 ± 1406.0	6122.1 ± 1008.0 b
P+DCD 25 mg L^–1^	3311.1 ± 514.3 c	274.7 ± 186.9 d	2000.2 ± 898.4 ab	228.7 ± 1006.1	2613.4 ± 713.4 c
P+OMW 50 mg L^–1^	6018.6 ± 2532.9 c	1812.3 ± 212.9 c	828.7 ± 421.0 c	96.3 ± 831.4	2651.1 ± 502.8 c
P+OMW 10 mg L^–1^	16541.5 ± 3957.1 b	3373.7 ± 1211.2 b	991.0 ± 210.1 c	11.1 ± 1525.3	5544.4 ± 1168.3 b
P+OMW 5 mg L^–1^	13787.2 ± 1611.0 b	3318.2 ± 641.7 b	1400.5 ± 431.9 bc	9.7 ± 725.1 Ns	5221.9 ± 492.6 b

a DCD at 25 mg/L (P+DCD) and OMW soluble fraction at 50, 10, and 5 mg of phenolic content/L (P+OMW), compared with control plants (Control P) and pots with soil but without plants (Control S) irrigated with distilled water. Values represent the mean ± standard deviation of 3 replicates. Values with different letters in the same column are significantly different (*p* < 0.05) according to Tukey’s HSD test. Ns, not significant.

P+DCD 25 mg•L^–1^ treatment exhibited a distinct N transformation pattern compared to Control P. Although NO_3_^–^ and NO_2_^–^ levels in soil at 31 DAP were not significantly different from Control P (*p* > 0.05), NH_4_^+^ retention was significantly higher (*p* < 0.05; Table 3), indicating partial inhibition of nitrification. This is consistent with the known action of DCD, which inhibits ammonia monooxygenase (AMO) in ammonia-oxidizing bacteria (AOB), delaying NH_4_^+^ oxidation to NO_2_^–^.^45^ The increased NH_4_^+^ in soil is attributable to both this inhibitory effect and the low C/N ratio (∼0.5) of DCD, which promotes mineralization of soil native ON. By virtue of its own chemical composition, DCD also directly contributes ON to the soil, leading to significantly higher ON and TN concentrations at 31 DAP compared to Control P. However, this higher NH_4_^+^ availability in soil led to increased NH_4_^+^ leaching, and although not statistically significant (*p* > 0.05), ON leaching also rose (Table 4).

In contrast, none of the treatments amended with P+OMW (50, 10, or 5 mg•L^–1^) showed significant differences (*p* > 0.05) in soil concentrations of NO_3_^–^, NO_2_^–^, NH_4_^+^, ON, or TN at 31 DAP when compared to Control P (Table 3). Unlike the DCD treatment, the P+OMW treatments did not result in higher NH_4_^+^ retention in the soil. This, however, does not rule out a potential nitrification-inhibiting effect. In fact, treatment P+OMW 50 mg•L^–1^ significantly reduced cumulative NO_3_^–^ leaching loads in leachates compared to Control P (*p* < 0.05; Table 4), indicating partial inhibition of nitrification at this dose. This effect was not observed in treatments P+OMW 10 and 5 mg•L^–1^. The absence of NH_4_^+^ accumulation in the P+OMW treatments is likely due to the lower input of ON from the soluble fraction of OMW,^25^ which limited its mineralization to NH_4_^+^. As a result, these treatments retained less NH_4_^+^ in the soil (Table 3) but also exhibited significantly lower cumulative NH_4_^+^ leaching loads in leachates than the P+DCD 25 mg•L^–1^ treatment (*p* < 0.05; Table 4), indicating a more balanced N transformation and a less pronounced disruption of the soil N cycle.

#### 3.2.2 Temporal Evolution and Cumulative Nitrogen Leaching under Different Treatments

As shown in Figures 2–4, the initial soil saturation followed by irrigation at 3 DAP led to substantial early leaching of inorganic nitrogen. These irrigations, necessary for uniform water distribution and seedling acclimation, caused significant NH_4_^+^ and NO_3_^–^ losses. In total, 72% and 82% of the cumulative NH_4_^+^ leaching occurred during these early events in Control S and Control P, respectively. Conversely, NO_3_^–^ losses represented only 9% and 12% of the total, while NO_2_^–^ remained negligible. Overall, these initial irrigations accounted for 22% and 26% of total inorganic N leaching in Control S and Control P, respectively.

**Figure 2:**
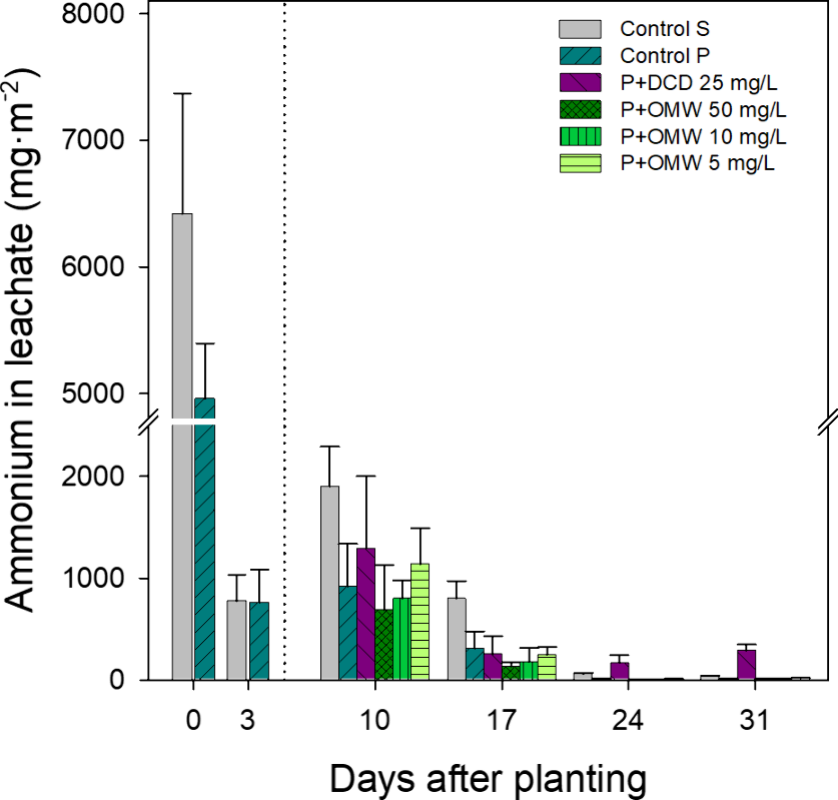
NH_4_^+^ levels (mg•m^–2^) in leachate water recorded on different evaluation days throughout the trial in pots with cultivated lettuce, irrigated with different nitrification inhibitor treatments: DCD at 25 mg/L (P+DCD) and OMW soluble fraction at 50, 10, and 5 mg of phenolic content/L (P+OMW), compared with control plants (Control P) and pots with soil but without plants (Control S). The dotted line represents the start of the treatments (5 DAP). Before the start of the treatments, as well as in the control pots, irrigation was performed using distilled water. Data represent the mean ± standard deviation of 3 replicates.

**Figure 3:**
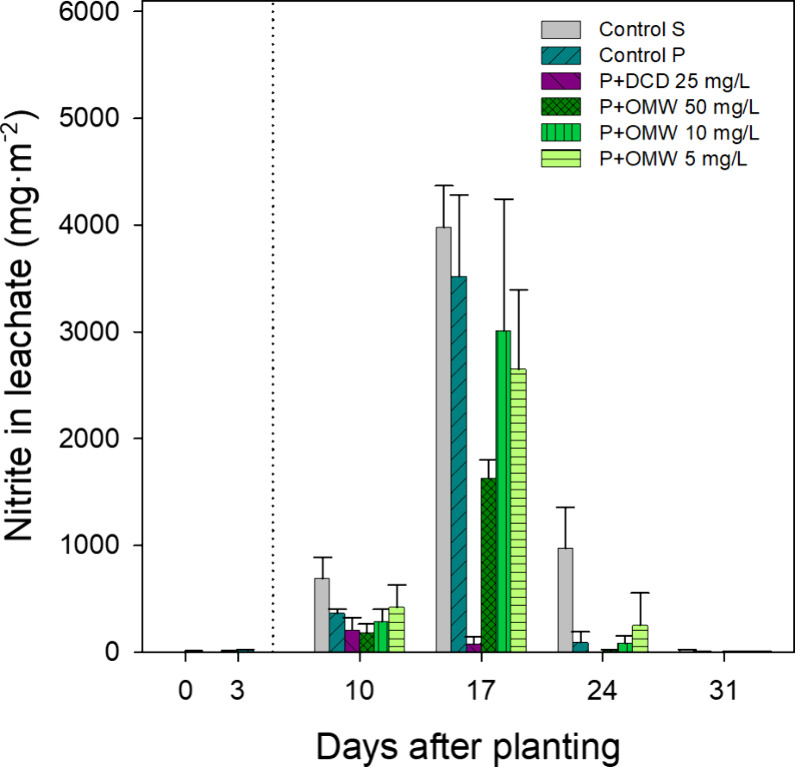
NO_2_^–^ levels (mg•m^–2^) in leachate water recorded on different evaluation days throughout the trial in pots with cultivated lettuce, irrigated with different nitrification inhibitor treatments: DCD at 25 mg/L (P+DCD) and OMW soluble fraction at 50, 10, and 5 mg of phenolic content/L (P+OMW), compared with control plants (Control P) and pots with soil but without plants (Control S). The dotted line represents the start of the treatments (5 DAP). Before the start of the treatments, as well as in the control pots, irrigation was performed using distilled water. Data represent the mean ± standard deviation of 3 replicates.

**Figure 4:**
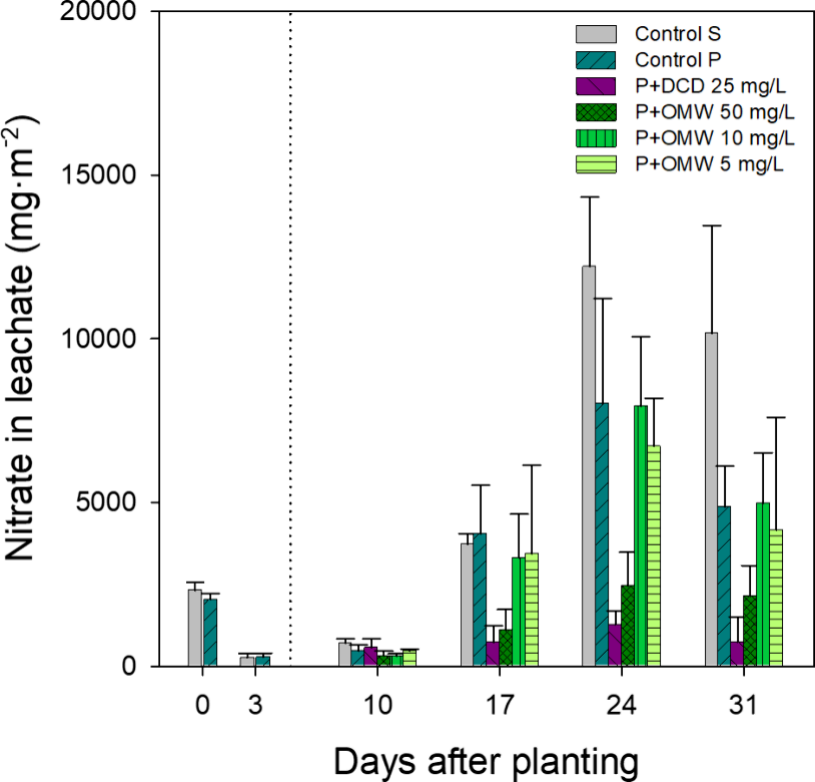
NO_3_^–^ levels (mg•m^–2^) in leachate water recorded on different evaluation days throughout the trial in pots with cultivated lettuce, irrigated with different nitrification inhibitor treatments: DCD at 25 mg/L (P+DCD) and OMW soluble fraction at 50, 10, and 5 mg of phenolic content/L (P+OMW), compared with control plants (Control P) and pots with soil but without plants (Control S). The dotted line represents the start of the treatments (5 DAP). Before the start of the treatments, as well as in the control pots, irrigation was performed using distilled water. Data represent the mean ± standard deviation of 3 replicates.

Although maintaining high soil moisture near field capacity is essential for optimal crop establishment and development,^46,47^ these early leaching pulses highlight a critical issue associated with incorporated fertilization strategies under real field conditions. Intense rainfall or excessive irrigation shortly after fertilization, but before plant uptake begins, can result in substantial NH_4_^+^ and NO_3_^–^ losses, increasing the risk of groundwater contamination.^48,49^ This risk is amplified in coarse or well-drained soils and underscores the limitations of uniform fertilization practices that disregard crop stage or weather conditions. Precision fertilization, which adjusts nutrient inputs spatially and temporally, is a more efficient and environmentally sound alternative.^50,51^

From 5 DAP onward, N leaching patterns in Control S and Control P reflected typical transformation dynamics of inorganic N in soil (Figures 2–4). In Control S, NH_4_^+^ leaching (Figure 2) was still noticeable at 10 DAP, decreased by 17 DAP, and was nearly absent by 24 and 31 DAP, indicating progressive nitrification. As NH_4_^+^ declined, NO_2_^–^ and NO_3_^–^ began to accumulate in the leachates. NO_2_^–^ concentrations were initially low but peaked sharply at 17 DAP (Figure 3), suggesting a transient accumulation phase. This behavior aligns with observations by Zhang et al.,^46^ who reported a distinct NO_2_^–^ peak under 60% field capacity, followed by rapid disappearance. The study confirmed that under high aeration and sufficient moisture, NO_2_^–^ may transiently accumulate as an intermediate during nitrification but does not persist. Consistent with that, NO_2_^–^ levels in our study declined by 24 DAP and were almost undetectable at 31 DAP. In contrast, NO_3_^–^ leaching (Figure 4) increased steadily from 10 DAP onward, peaked at 24 DAP, and remained high at 31 DAP, indicating ongoing nitrification in the absence of effective sinks such as plant uptake or microbial immobilization. This sustained NO_3_^–^ presence underscores the environmental risk of nitrate accumulation in bare soils.

In Control P, the presence of *Lactuca sativa L*. modulated N dynamics. NH_4_^+^ leaching (Figure 2) peaked at 10 DAP and dropped sharply thereafter, with cumulative NH_4_^+^ losses reduced by 55% compared to Control S (Table 4), suggesting active plant N uptake. NO_2_^–^ followed a similar pattern, peaking at 17 DAP (Figure 3) but showing 30% lower total leaching than in Control S (Table 4), likely due to reduced N availability caused by plant N uptake. By 24 DAP, NO_2_^–^ was nearly absent. NO_3_^–^ leaching also increased over time (Figure 4) but was 35% lower than in Control S (Table 4), likely due to plant assimilation of both NH_4_^+^ and NO_3_^–^, which limited nitrification and associated losses.

The temporal evolution of NH_4_^+^ leaching in P+DCD 25 mg/L treatment showed distinct trends compared to Control P. Although NH_4_^+^ levels at 10 DAP were higher in P+DCD 25 mg/L, differences were not statistically significant (*p* > 0.05; Figure 2). A slight decrease was observed at 17 DAP, yet P+DCD 25 mg/L was the only treatment that still showed detectable NH_4_^+^ leaching at 24 and 31 DAP. By inhibiting nitrification, DCD likely increased NH_4_^+^ retention in soil and delayed its oxidation to NO_2_^–^, thereby prolonging its presence in the soil solution and explaining the detectable NH_4_^+^ leaching observed at 24 and 31 DAP. However, beyond its inhibitory action, DCD also introduces ON with a very low C/N ratio (∼0.5), which may stimulate mineralization processes. This mechanism was previously observed in plant-free soils, where DCD stimulated mineralization and NH_4_^+^ accumulation in soil.^25^ Moreover, Gioacchini et al.^52^ reported that DCD, when applied with N-(*n*-butyl) thiophosphoric triamide (NBPT), a urease inhibitor, increased nitrogen losses through NH_3_ volatilization and NO_3_^–^ leaching. These effects were attributed to NH_4_^+^ retention in soil by DCD, which likely induced a priming effect that accelerated the mineralization of native soil organic matter and released additional nitrogen. Overall, P+DCD 25 mg/L resulted in 59% more cumulative NH_4_^+^ leaching than Control P (Table 4).

In contrast, the P+OMW 50 mg/L treatment showed a pattern similar to Control P: a peak at 10 DAP followed by a sharp decline, with NH_4_^+^ leaching suppressed from 24 DAP onward (Figure 2). Despite nonsignificant differences, NH_4_^+^ leaching concentrations were consistently lower than in Control P, leading to a 34% reduction in total NH_4_^+^ leached (Table 4).

Regarding NO_2_^–^, P+DCD 25 mg/L lacked the transient 17 DAP peak observed in Control P and other treatments. Instead, a maximum was detected at 10 DAP, followed by a continuous decline to undetectable levels by 24 DAP (Figure 3), consistent with AMO inhibition by DCD.^45^ The P+OMW 50 mg/L treatment displayed a temporal pattern similar to that of Control P but with significantly lower NO_2_^–^ leaching concentrations at all sampling points (*p* < 0.05), suggesting effective nitrification inhibition. Overall, cumulative NO_2_^–^ leaching was reduced by 93% with DCD and by 54% with OMW soluble fraction compared to Control P (Table 4).

Both treatments also reduced NO_3_^–^ leaching compared to Control P. While P+DCD 25 mg/L achieved an 81% reduction versus 65% with P+OMW 50 mg/L, differences were not statistically significant (Table 4). The response observed under the P+OMW 50 mg/L treatment may be related to a combined effect of the phenolic concentration applied and the high TOC/TN mass ratio of the OMW soluble fraction (Table 1), which may jointly contribute to nitrification inhibition in soil and to microbial immobilization of inorganic N. Similar effects have been observed in soils treated with OMW soluble fraction,^25^ as well as with other organic amendments rich in carbon and phenolic compounds, such as *Arctostaphylos uva-ursi* extracts, which have been reported to inhibit nitrification and promote microbial nitrogen immobilization.^53^ Karpouzas et al.^54^ reported that repeated OMW application caused a drastic reduction in mineral N and altered ammonia-oxidizing bacterial communities in two contrasting soils, namely a loamy sand and a sandy loam. In the OMW soluble fraction used in the present study, hydroxytyrosol and tyrosol were the main quantified phenolic compounds, whereas oleuropein was below the detection limit (Table 1), in agreement with phenolic profiles commonly reported for OMW.^24,55^ Previous work has also shown that polyphenols can suppress soil nitrification by reducing the abundance of microorganisms carrying nitrification-related functional genes,^56^ while individual BNI-related phenolic compounds such as caffeic acid and vanillic acid can inhibit ammonia oxidizers and suppress soil nitrification.^57^ Likewise, Tsiknia et al.^58^ observed lower NO_3_^–^-N concentrations in soil after OMW application and explicitly attributed the initial decline to immobilization, in parallel with reduced *amoA* gene copies of archaeal and bacterial ammonia oxidizers during the first days after application. Taken together, the lower NO_2_^–^ and NO_3_^–^ leaching observed under P+OMW 50 mg•L^–1^ (Figure 5) is consistent with a combined response involving phenolic-associated suppression of nitrification and carbon-driven microbial immobilization of mineral N. Although hydroxytyrosol and tyrosol were the most abundant quantified phenolics in the OMW soluble fraction, the present results do not allow the effects of individual compounds on N transformations to be disentangled. These results demonstrate the comparable inhibitory potential of OMW soluble fraction and DCD at these concentrations, supporting the viability of natural, circular alternatives for N management.

**Figure 5:**
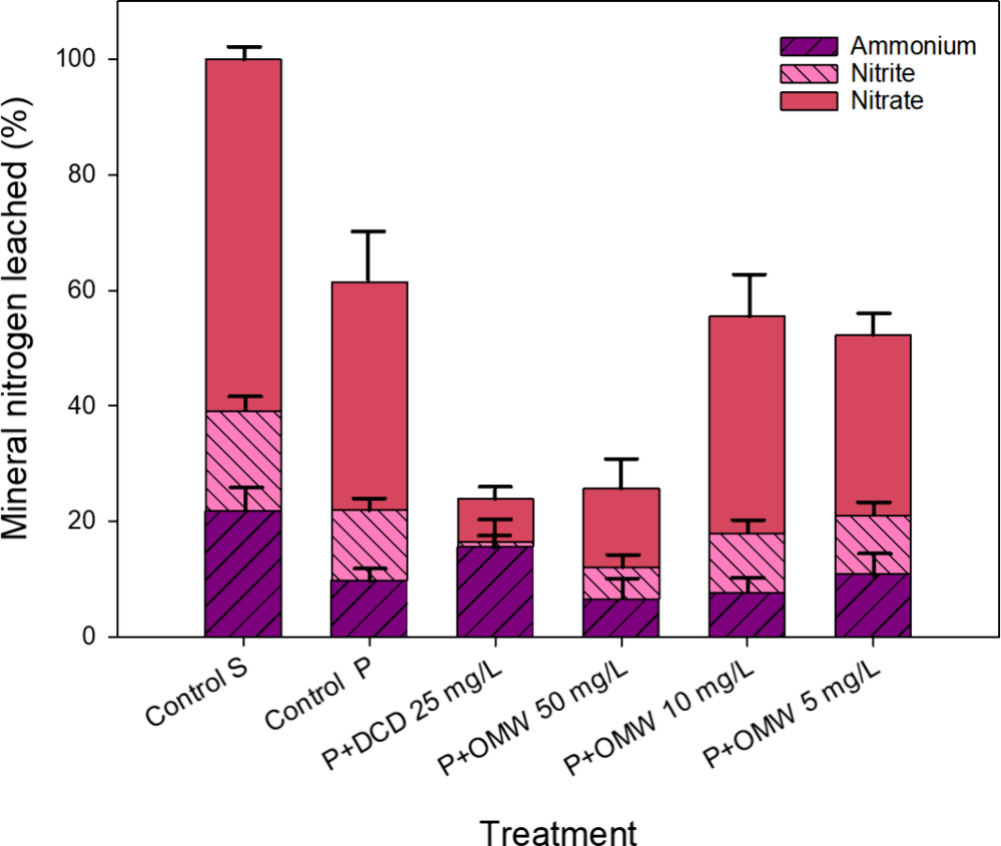
Data of the quantitative contribution of different nitrogen species (NH_4_^+^, NO_2_^–^ and NO_3_^–^) to the mineral nitrogen input in leachate water (%) from 5 DAP to 31 DAP in pots with cultivated lettuce, irrigated with different nitrification inhibitor treatments: DCD at 25 mg/L (P+DCD) and OMW soluble fraction at 50, 10, and 5 mg of phenolic content/L (P+OMW), compared with control plants (Control P) and pots with soil but without plants (Control S) irrigated with distilled water. Values represent the mean ± standard deviation of 3 replicates.

Finally, at lower phenolic doses (P+OMW 10 mg/L and 5 mg/L), the temporal patterns of NH_4_^+^, NO_2_^–^, and NO_3_^–^ leaching closely resembled those of Control P, with no significant differences in leached concentrations (Figures 2–4) or cumulative values (Table 4). These findings indicate that such low OMW soluble fraction concentrations were insufficient to inhibit nitrification under the conditions tested.

Regarding cumulative leaching of total inorganic nitrogen throughout the cultivation period (Figure 5), Control P significantly reduced N losses by 39% compared to Control S (*p* < 0.05), underscoring the beneficial effect of plant presence on limiting inorganic N leaching. When compared to Control P, P+OMW 10 mg/L and P+OMW 5 mg/L decreased total inorganic nitrogen leaching losses by 9% and 15%, respectively, although these reductions were not statistically significant (*p* < 0.05). Among all treatments, only P+DCD 25 mg/L and P+OMW 50 mg/L achieved statistically significant reductions (*p* < 0.05) in cumulative total inorganic nitrogen leaching relative to Control P.

As previously discussed, while P+DCD 25 mg/L was more effective than P+OMW 50 mg/L in reducing NO_2_^–^ and NO_3_^–^ leaching compared to Control P—by 93% and 81%, respectively, versus 54% and 65%—it also led to 59% higher NH_4_^+^ leaching. In contrast, P+OMW 50 mg/L reduced NH_4_^+^ leaching by 34% compared to Control P. Consequently, the total inorganic nitrogen leached under both treatments was comparable, with overall reductions of 61% (P+DCD 25 mg/L) and 58% (P+OMW 50 mg/L) relative to Control P, with no statistically significant difference between them (*p* < 0.05). These laboratory findings align with field data reported by Cameron et al.,^59^ who observed that DCD application—at a rate of 10 kg•ha^–1^—consistently reduced NO_3_^–^ leaching by 48–69% when applied with animal urine in April, highlighting the reproducibility of its inhibitory effect across different experimental conditions. Moreover, a meta-analysis by Cai et al.^60^ confirmed that on average, DCD decreased NO_3_^–^ losses by 46%, while NBPT+DCD achieved a 42% reduction. These effects were independent of study type (laboratory, greenhouse, in situ field, or lysimeter) and duration (≤180 vs >180 days). The analysis also indicated that the effectiveness of DCD was greater when applied during autumn or winter and improved with increasing application rates—being significantly more effective at >30 kg•ha^–1^ compared to lower doses. However, application rates above 20 kg•ha^–1^ did not lead to increased plant N uptake, suggesting a trade-off between NO_3_^–^ leaching control and N availability for crops.^60^

These findings demonstrate that natural inhibitors derived from OMW soluble fraction, when applied at an adequate concentration, can achieve similar effectiveness to that of the synthetic nitrification inhibitor DCD in reducing total inorganic nitrogen leaching losses. Furthermore, it represents a more sustainable alternative, as concerns have been raised about the environmental persistence of DCD and other synthetic inhibitors. These compounds have been found to leach into water bodies, persist in soils, and even accumulate in the food chain—having been detected in aquatic organisms and dairy products such as milk.^19^

### 3.3 Growth Performance and Quality Traits of *Lactuca sativa L*. Cultivated under Different Treatments

At 31 DAP, lettuce plants under P+DCD 25 mg/L and P+OMW 50 mg/L exhibited greater visual development compared to the other treatments (Figure 6A), a trend corroborated by the fresh weight data (Figure 6B). Both treatments significantly increased fresh biomass relative to Control P (*p* < 0.05), with gains of 54% and 56%, respectively, and no significant differences between them.

**Figure 6:**
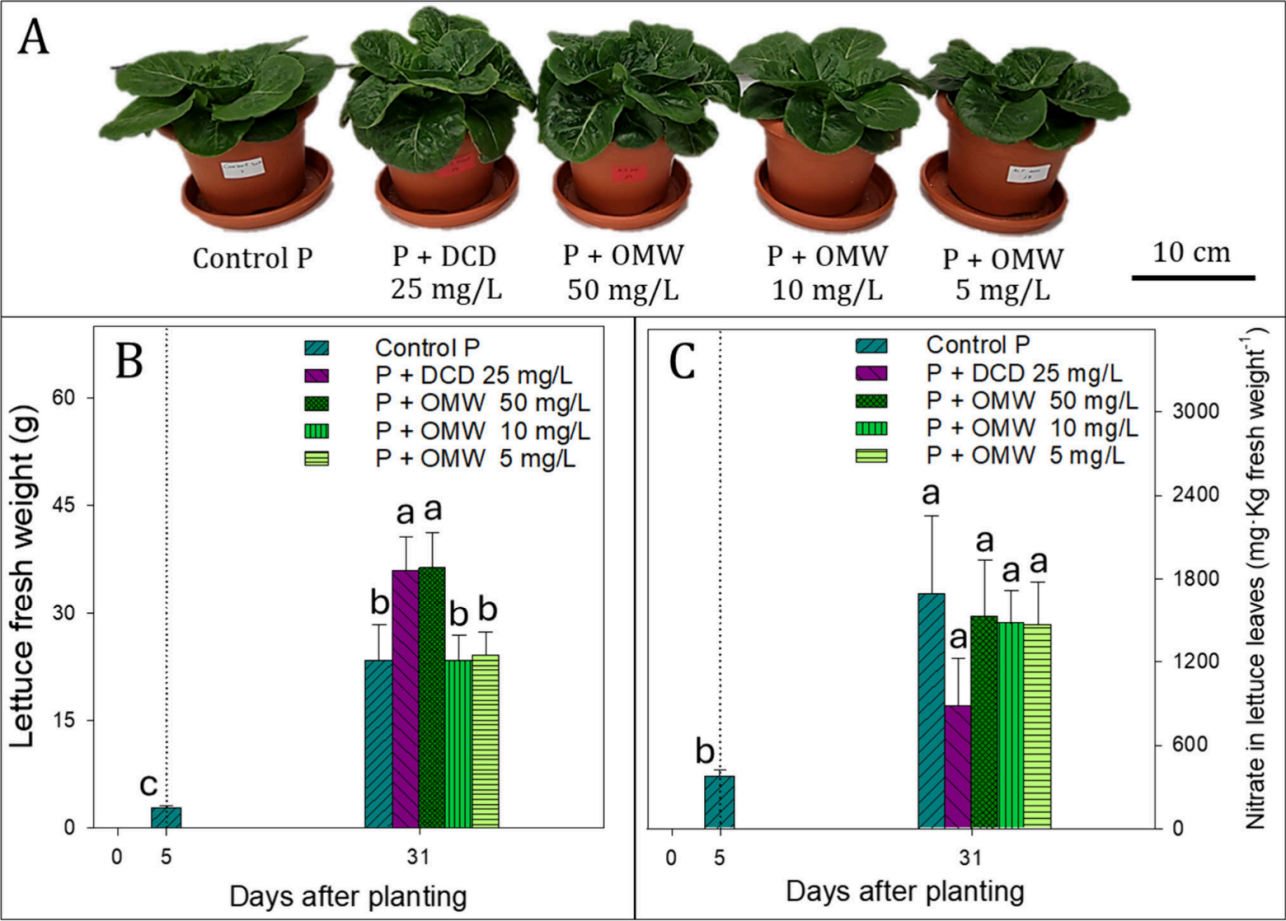
Plant growth characteristics (A), fresh weight per plant (g) (B) and NO_3_^–^ level in leaves (mg•kg^–1^ fresh weight) (C) of lettuce irrigated with different nitrification inhibitor treatments: DCD at 25 mg/L (P+DCD) and OMW soluble fraction at 50, 10, and 5 mg of phenolic content/L (P+OMW), compared with control plants (Control P). The dotted line represents the start of the treatments applied via fertigation. Before the start of the treatments, as well as in the control pots, irrigation was performed using distilled water. Bars are mean ± standard deviation of 3 replicates. Bars with different letters are significantly different (*p* < 0.05) according to Tukey’s HSD test.

These results indicate that the application of OMW soluble fraction at 50 mg/L phenolic content does not exert phytotoxic effects commonly associated with higher doses of olive-derived residues, particularly OMW.^22,26^ On the contrary, it promoted lettuce growth comparable to the synthetic inhibitor DCD. This beneficial effect may derive from both enhanced NUE due to nitrification inhibition and supplementary inputs of ON. In the case of OMW soluble fraction, its macro- and micronutrient content may also contribute positively.^24^

This nutrient input is especially relevant considering the early nitrogen losses described previously (Figures 2–4). The saturation of the soil system followed by control irrigation at 3 DAP resulted in substantial leaching of inorganic nitrogen—particularly NH_4_^+^—before effective plant uptake was established. In Control P, 82% of total NH_4_^+^ leaching occurred during these early irrigation events, contributing to 26% of the total inorganic N losses over the experiment. Thus, the nutrient contributions from DCD and OMW soluble fraction may help mitigate early stage deficits. Control P produced a mean fresh weight of 23.3 ± 5 g, below values reported for multileaf *Lactuca sativa L*. under optimal conditions,^12^ whereas the improved growth in P+DCD 25 mg/L and P+OMW 50 mg/L treatments enabled the crop to reach sizes comparable to those previously reported under favorable conditions. These results are particularly relevant under field conditions prone to high leaching or precipitation.

Lower concentrations of OMW soluble fraction (10 and 5 mg/L) did not produce significant changes in fresh weight compared to Control P (*p* < 0.05), indicating limited efficacy in promoting growth or inhibiting nitrification. This trend was consistent across additional morphological parameters, with no significant differences observed in leaf area, length, or width (Table 5), suggesting a negligible impact on plant development.

**Table 5:** Morphological Characteristics (Area, Length, and Width) and Color Attributes (Hue, Saturation, and Brightness) of the Leaves in Cultivated Lettuce Irrigated with Different Nitrification Inhibitor Treatments^a^

Treatment	Leaf area (cm^2^)	Leaf length (cm)	Leaf width (cm)	Color HUE (0–255)	Color saturation (0–255)	Brightness (0–255)
Control P	42.54 ± 6.77 b	9.92 ± 0.65 b	6.26 ± 0.55 b	60.99 ± 0.18	162.82 ± 4.12 b	117.62 ± 0.56
P+DCD 25 mg L^–1^	55.27 ± 4.24 a	11.12 ± 0.38 a	7.16 ± 0.38 a	60.48 ± 0.72	175.96 ± 1.61 a	113.98 ± 5.61
P+OMW 50 mg L^–1^	52.23 ± 4.85 a	10.66 ± 0.21 a	7.05 ± 0.45 a	61.68 ± 0.41	168.36 ± 5.42 ab	106.99 ± 1.58
P+OMW 10 mg L^–1^	40.80 ± 7.13 b	9.63 ± 0.87 b	6.08 ± 0.49 b	61.79 ± 0.63	159.05 ± 4.39 b	110.65 ± 2.85
P+OMW 5 mg L^–1^	41.09 ± 5.72 b	9.60 ± 0.50 b	6.16 ± 0.35 b	61.28 ± 0.45 ns	154.92 ± 9.25 b	119.35 ± 3.42 ns

a DCD at 25 mg/L (P+DCD) and OMW soluble fraction at 50, 10, and 5 mg of phenolic content/L (P+OMW), compared with control plants (Control P) irrigated with distilled water. Values represent the mean ± standard deviation of 3 replicates at final harvest time (31 days after planting). Values with different letters in the same column are significantly different (*p* < 0.05) according to Tukey’s HSD test.

Across all treatments, leaf count and shape—as measured by roundness—did not differ significantly (data not shown), indicating that differences in biomass were primarily attributable to variations in individual leaf dimensions rather than number or form.

In contrast, P+DCD 25 mg/L and P+OMW 50 mg/L treatments significantly increased leaf area, length, and width compared to Control P (*p* < 0.05; Table 5; Figure S1). Leaf length increased by 12% and 7%, respectively, slightly exceeding the average values (∼10.5 cm) previously reported for multileaf *Lactuca sativa L*. under optimal conditions.^4^

Regarding leaf color attributes—key parameters for consumer preference and visual appeal in leafy vegetables—no significant differences (*p* < 0.05) were observed among treatments in hue or brightness (Table 5). Only P+DCD 25 mg/L showed a significantly higher color saturation compared to Control P, while P+OMW 50 mg/L displayed a slightly elevated, though not significant, value. Lower OMW soluble fraction concentrations (10 and 5 mg/L) had no significant effect. These results are relevant as color plays a critical role in the selection and acceptance of fresh salad crops,^61^ with green color intensity linked to chlorophyll content and the perception of freshness and visual quality.^12,62^ Therefore, the maintenance of typical green coloration—especially under P+OMW 50 mg/L—indicates that this amendment does not compromise visual quality, essential for marketability.

NO_3_^–^ profiles in lettuce leaves showed a significant increase (*p* < 0.05) across all treatments at 31 DAP compared to 5 DAP (Figure 6C). Among treatments, only P+DCD 25 mg/L resulted in a notably lower leaf NO_3_^–^ content on a fresh-weight basis—48% less than Control P—although this difference was not statistically significant (Figure 6C). Lettuce plants grown under OMW soluble fraction treatments showed leaf NO_3_^–^ contents comparable to those of Control P. These results suggest that DCD may help reduce NO_3_^–^ accumulation in edible tissues, potentially lowering health risks for consumers. However, it is important to consider the potential drawbacks associated with DCD. As previously discussed, DCD residues may persist in soils, leach into water bodies, and even bioaccumulate through the food chain, with documented detections in aquatic organisms and dairy products.^19^ Padash et al.^63^ further warn that high concentrations of DCD can impair chlorophyll content and plant growth in *Lactuca sativa L.*, potentially causing phytotoxicity or entering the food chain unless mitigated by beneficial plant–fungus interactions.

In contrast, leaf NO_2_^–^ concentrations remained extremely low across all treatments, consistently below 1 mg•kg^–1^ fresh weight, with no statistically significant differences (*p* < 0.05) compared to Control P (data not shown). Moreover, NO_2_^–^ levels in leafy vegetables are currently unregulated under European legislation.

On the other hand, despite not reducing leaf NO_3_^–^ accumulation as effectively as DCD, P+OMW 50 mg/L allowed for the production of lettuces that meet food safety and quality standards. According to Commission Regulation (EU) No 1258/2011—which establishes maximum NO_3_^–^ levels in leafy vegetables—the most restrictive threshold for *Lactuca sativa L*. is 3000 mg NO_3_^–^•kg^–1^ fresh weight for crops harvested between April and September under open-field conditions. Lettuce leaves from all treatments in this study, including P+OMW 50 mg/L, showed NO_3_^–^ contents on a fresh-weight basis well below this regulatory limit, indicating their suitability for safe commercial production. Importantly, this treatment achieved compliance without the potential environmental and toxicological risks associated with synthetic inhibitors, supporting its value as a sustainable alternative.

## 4 Conclusions

This study provides new insights into the sustainable use of OMW soluble fraction as a natural nitrification inhibitor in leafy vegetable production. The novelty of this work lies in providing the first experimental evidence that low concentrations of OMW soluble fraction can serve as an effective natural alternative to synthetic inhibitors in lettuce cultivation, combining fertilization and environmental protection within a circular economy framework. At a phenolic concentration of 50 mg/L, OMW soluble fraction enhanced nitrogen retention, supported biomass production, and preserved quality traits in *Lactuca sativa* L. without inducing phytotoxic effects. The performance of this treatment was comparable to that of the synthetic inhibitor DCD. Lower OMW soluble fraction doses (5–10 mg/L) were ineffective in improving nitrogen use or crop performance.

Importantly, all treatments produced lettuce leaves with NO_3_^–^ contents on a fresh-weight basis below the EU safety limits, confirming their suitability for commercial use. While DCD slightly reduced leaf NO_3_^–^ concentration, plants grown under OMW soluble fraction treatments maintained acceptable levels without compromising visual or nutritional quality. These results position low-dose OMW soluble fraction as a viable, circular alternative for mitigating nitrogen losses and improving crop productivity under leaching-prone conditions.

In addition, this work reveals a previously unreported analytical limitation: strong matrix interference in HPLC-UV/vis-based NO_2_^–^ quantification in lettuce leaf extracts. A coeluting peak with high absorbance at 275 nm compromised detection at 220 nm, resulting in poor agreement with colorimetric Griess assay data. This highlights the need for method validation and cleanup strategies when analyzing NO_2_^–^ in leafy vegetable tissues, particularly under low-concentration conditions.

However, although the observed effects are consistent with previously reported mechanisms, such as the role of phenolic compounds in inhibiting nitrification and the promotion of microbial nitrogen immobilization associated with high TOC/TN ratios, these processes were not directly quantified in this study. Further research, including direct quantification of the processes involved, would help to confirm the underlying mechanisms.

Overall, the findings support the dual environmental and agronomic value of OMW valorization and contribute new evidence for improving analytical reliability in NO_3_^–^/NO_2_^–^ monitoring in plant matrices.

## Supplementary Material



## Data Availability

Data supporting the reported results can be provided upon reasonable request to the corresponding author, where appropriate.

## Notes

Images showing leaf samples of lettuce (*Lactuca sativa L*. type multileaf) cultivated under different nitrification inhibitor treatments at final harvest (PDF)
