# Supplementary material for: Olive Mill Wastewater: A Sustainable Natural Solution for Nitrate Contamination Control in Agricultural Systems and Quality Enhancement of Multileaf Lettuce Cultivation
Source: ACS Sustain Chem Eng. 2026 Apr 27;14(18):8931–44. doi: 10.1021/acssuschemeng.6c02426 (PMC13170604; doi:10.1021/acssuschemeng.6c02426)

Supporting Information

Olive mill wastewater: a sustainable natural solution for nitrate contamination control in agricultural systems and quality enhancement of multi-leaf lettuce cultivation

Adrián Hernández Fernández[1], Eduardo Iniesta Lopez[1], Yolanda Garrido[1], Ana Sánchez Zurano[1*], Antonia Pérez de los Ríos[1], Francisco José Hernández Fernández[1]

[1]Department of Chemical Engineering, University of Murcia, Murcia, 30100, Spain

*Corresponding author: Ana Sánchez Zurano

Email: azurano@um.es

Phone: +34 868 887 358

Number of pages: 2

Number of figures: 1

Number of tables: 0

Figure S1. Representative leaf samples of lettuce (*Lactuca sativa L.* type multi-leaf) cultivated under different nitrification inhibitor treatments at final harvest (31 days after planting). Images correspond to: (A) Control P (distilled water), (B) P+DCD 25 mg/L, (C) P+OMW 50 mg phenolic content/L, (D) P+OMW 10 mg phenolic content/L, and (E) P+OMW 5 mg phenolic content/L. Leaves were selected to visually illustrate differences in size and morphology among treatments.

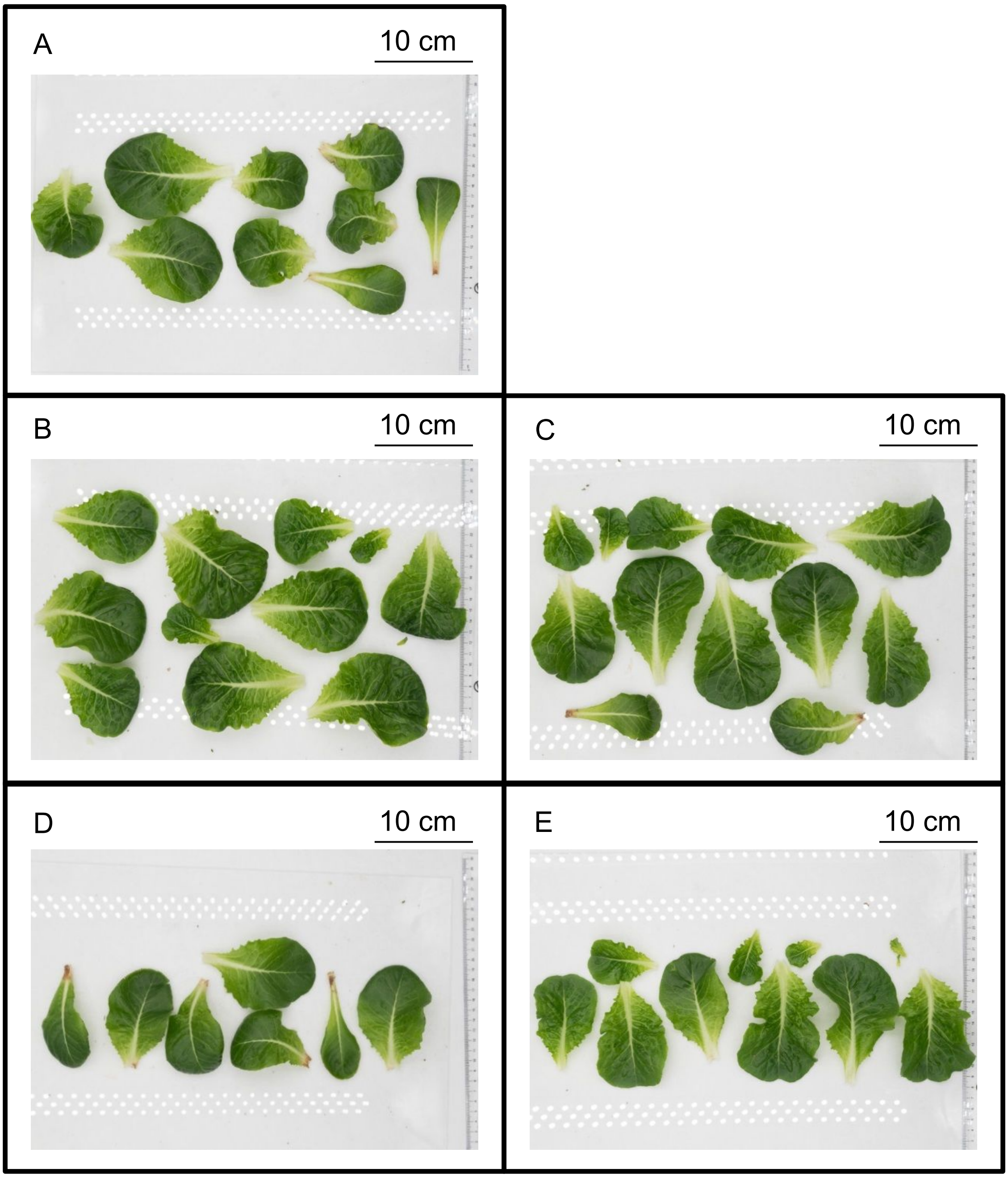

Supplement: Supplementary file 1 [file sc6c02426_si_001.pdf]
